# Omics sciences for cervical cancer precision medicine from the perspective of the tumor immune microenvironment

**DOI:** 10.32604/or.2024.053772

**Published:** 2025-03-19

**Authors:** GUANTING PANG, YAOHAN LI, QIWEN SHI, JINGKUI TIAN, HANMEI LOU, YUE FENG

**Affiliations:** 1 College of Pharmaceutical Science, Zhejiang University of Technology, Hangzhou, 310014, China; 2 College of Artificial Intelligence and Big Data for Medical Sciences, Shandong First Medical University & Shandong Academy of Medical Sciences, Jinan, 250000, China; 3 Collaborative Innovation Center for Green Pharmaceuticals, Zhejiang University of Technology, Hangzhou, 310014, China; 4 Hangzhou Institute of Medicine (HIM), Chinese Academy of Sciences, Hangzhou, 310022, China; 5 Department of Gynecological Oncology, Zhejiang Cancer Hospital, Hangzhou, 310022, China

**Keywords:** Omics, Tumor immune microenvironment (TIME), Precision medicine, Cervical cancer (CC)

## Abstract

Immunotherapies have demonstrated notable clinical benefits in the treatment of cervical cancer (CC). However, the development of therapeutic resistance and diverse adverse effects in immunotherapy stem from complex interactions among biological processes and factors within the tumor immune microenvironment (TIME). Advanced omic technologies offer novel insights into a more expansive and thorough layer of the TIME. Furthermore, integrating multidimensional omics within the frameworks of systems biology and computational methodologies facilitates the generation of interpretable data outputs to characterize the clinical and biological trajectories of tumor behavior. In this review, we present advanced omics technologies that utilize various clinical samples to address scientific inquiries related to immunotherapies for CC, highlighting their utility in identifying metastasis dissemination, recurrence risk, and therapeutic resistance in patients treated with immunotherapeutic approaches. This review elaborates on the strategy for integrating multi-omics data through artificial intelligence algorithms. Additionally, an analysis of the obstacles encountered in the multi-omics analysis process and potential avenues for future research in this domain are presented.

## Introduction

Cervical cancer (CC) is one of the most common gynecologic tumors, primarily caused by persistent human papillomavirus (HPV) infection. Unlike other tumors, CC involves a long process of precancerous lesions, making it a preventable and therapeutically diverse cancer. In recent years, the use of immune checkpoint inhibitors (ICIs) has led to a paradigm shift in therapeutic approaches, demonstrating remarkable clinical efficacy in the management of CC [1]. Currently, immunotherapy has emerged as a highly effective anti-tumor strategy, leading to a shift in the traditional CC treatment approach, which mainly relied on surgery and chemoradiotherapy. The National Comprehensive Cancer Network guidelines now endorse immunotherapy as a primary and secondary treatment for recurrent or metastatic CC, also showing notable efficacy in locally advanced cases. However, despite numerous clinical and preclinical studies, pembrolizumab remains the only Programmed Cell Death Protein 1 (PD-1) inhibitor approved for CC patients. Studies indicate clear benefits of combining ICIs with platinum-based chemotherapy in treating this disease [2]. The Keynote-158 [3], Keynote-826 [4], EMPOWER-Cervical 1 (NCT03257267), and Keynote-028 [5] trials have demonstrated that PD-1 inhibitors, when combined with chemotherapy, are more effective than chemotherapy alone in CC patients. Immunotherapy addresses many of the limitations of surgery and chemoradiotherapy, leading to significant improvements in overall survival (OS). Tewari’s study highlighted that cemiplimab achieved significant survival extension compared to single-agent chemotherapy in patients with recurrent CC after initial treatment with platinum-based chemotherapy [6]. Despite the significant advances in immunotherapy, particularly with ICIs, for CC, treatment responses can vary significantly among patients. Therefore, before initiating immunotherapy, it is crucial to identify effective and reliable biomarkers for screening. This helps determine the optimal timing for immunotherapy and which patients are most likely to benefit from it. Current biomarkers like PD-1 and Programmed Cell Death Ligand 1 (PD-L1) have some predictive value, but their sensitivity and specificity are relatively low.

**Figure 1:**
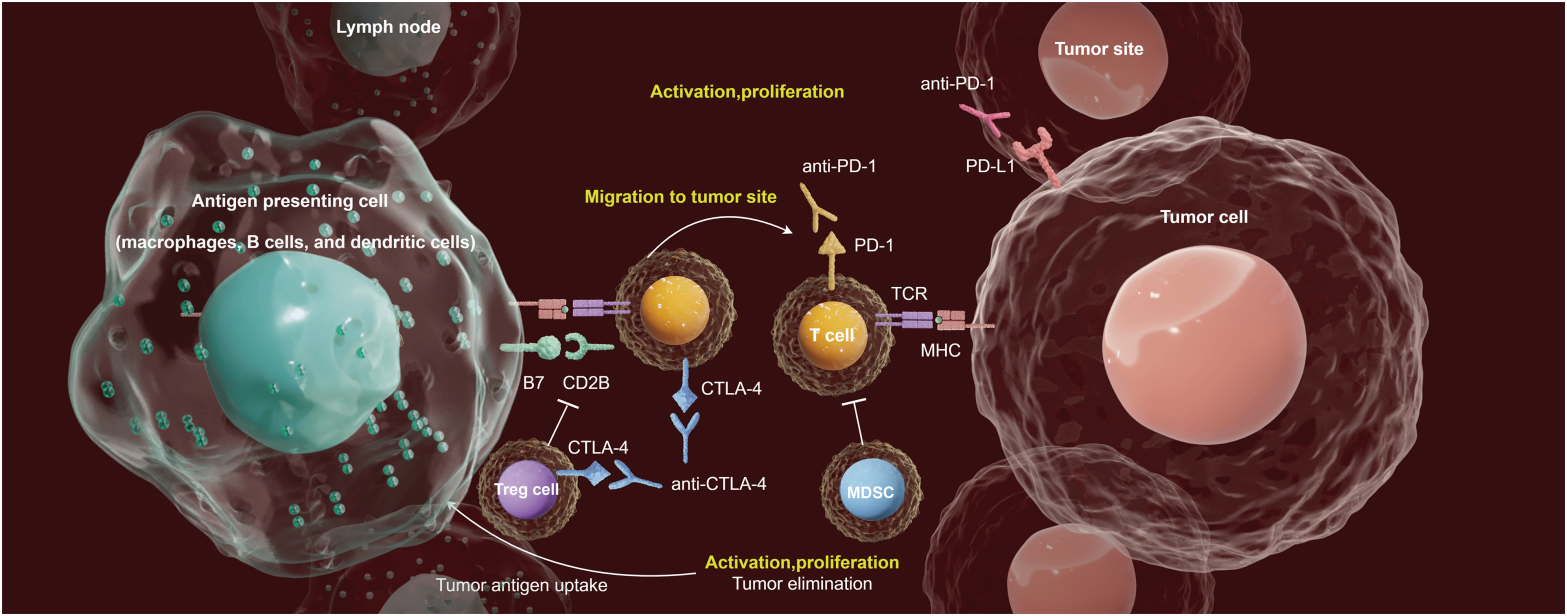
Emerging immunotherapy strategies adapted to the TlME in CC. The antigen-presentation (by the antigen-presenting cells, such as macrophages, B cells, and dendritic cells) in tumor-draining lymph nodes activates T-cell. Activated T-cells help antibody class-switching, and production of tumor-specific antibodies. ICI treatments (Anti-PD-1, Anti-PD-L1 and Anti-CTLA-4 therapies) leverages the TME to suppress the immune evasion mechanisms of tumor cells, thus providing opportunities for the development of novel therapeutic strategies.

The tumor immune microenvironment (TIME) plays a crucial role in immunotherapies for patients with CC [7]. The intricate and diverse ecosystems within the tumor microenvironment (TME) significantly influence the interactions and behaviors of malignant cells with other cellular populations, which is essential for tumor progression and the response to immunotherapy (Fig. 1). Immune cells and their secreted factors are key components of the TME, with cytotoxic CD8^+^ T-cells being primed by various antigen-presenting cells, such as macrophages, B cells, and dendritic cells, to regulate the cytotoxic effector T-cell response. Additional prominent populations that contribute to a protumorigenic response include myeloid-derived suppressor cells and immunomodulatory regulatory T-cells, resulting in immunosuppression and resistance to immunotherapy [8]. Monitoring tumor-infiltrating Tregs in CC may provide valuable insights, as peripheral Tregs can be recruited to tumor sites and proliferate through self-antigen recognition by memory Tregs. In a separate study conducted by our research group, it was postulated that during the early stages of HPV infection in CCs, plasmacytoid dendritic cells (pDCs) might have exerted an anti-HPV function for a short period [9]. However, as HPV persists and leads to malignant transformation of epithelial cells, pDCs may have transitioned to a state of immune anergy and developed immune tolerance. Based on the above information, we focused on the TIME as a critical determinant of immunotherapy in CC.

**Figure 2:**
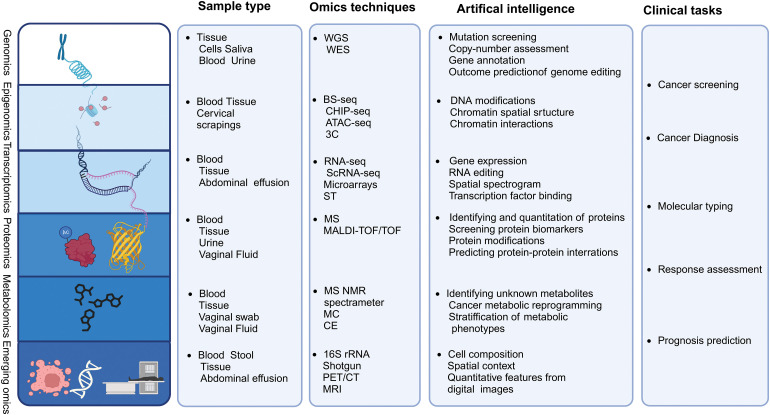
Multi-omics technologies. Tissue and body fluid samples can be analyzed using multi-omics platforms, including genomics, transcriptomics, epigenomics, proteomics, metabolomics, metagenomics, single-cell omics, and radiomics, using artificial intelligence algorithms for data integration. Additionally, the current clinical challenges that omics research may encounter are summarized.

The rapid advancement of high-throughput sequencing technologies and the evolution of large-scale computational analyses, known as omics, have significantly enhanced our understanding of intratumoral heterogeneity in oncology [10]. The continuous improvement of biotechnological omics technologies has enabled researchers to access information at various levels by utilizing omics data obtained from clinical samples, thereby revealing previously unknown immune states of tumors regarding immune recognition or immune ignorance [11] (Fig. 2). An emerging trend in this field involves using multi-omics data analysis to thoroughly understand the complex interplay between various layers of molecules and the estimated heritability of diseases. Integrating multi-omics data for immune characterization has significantly advanced the validation of potential therapeutic biomarkers, thus enabling the potential for personalized treatment with ICIs basis of the tumor immune profile [12].

This review examines recent advances in multi-omics research to explore the interplay and temporal changes within the TIME in gynecological malignancies. Furthermore, this paper explores the use of multi-omics technology to understand intricate regulatory networks and immune diversity. Additionally, we provide a comprehensive summary of the opportunities and obstacles of multi-omics analysis in the context of gynecological tumors and future prospects for their integration. The incorporation of multi-omics dimensions is anticipated to propel advances in immunomics biology research and facilitate the clinical translation of CC.

## Application of Multidimentional Omics to the TME of CC

### Genomics

**Figure 3:**
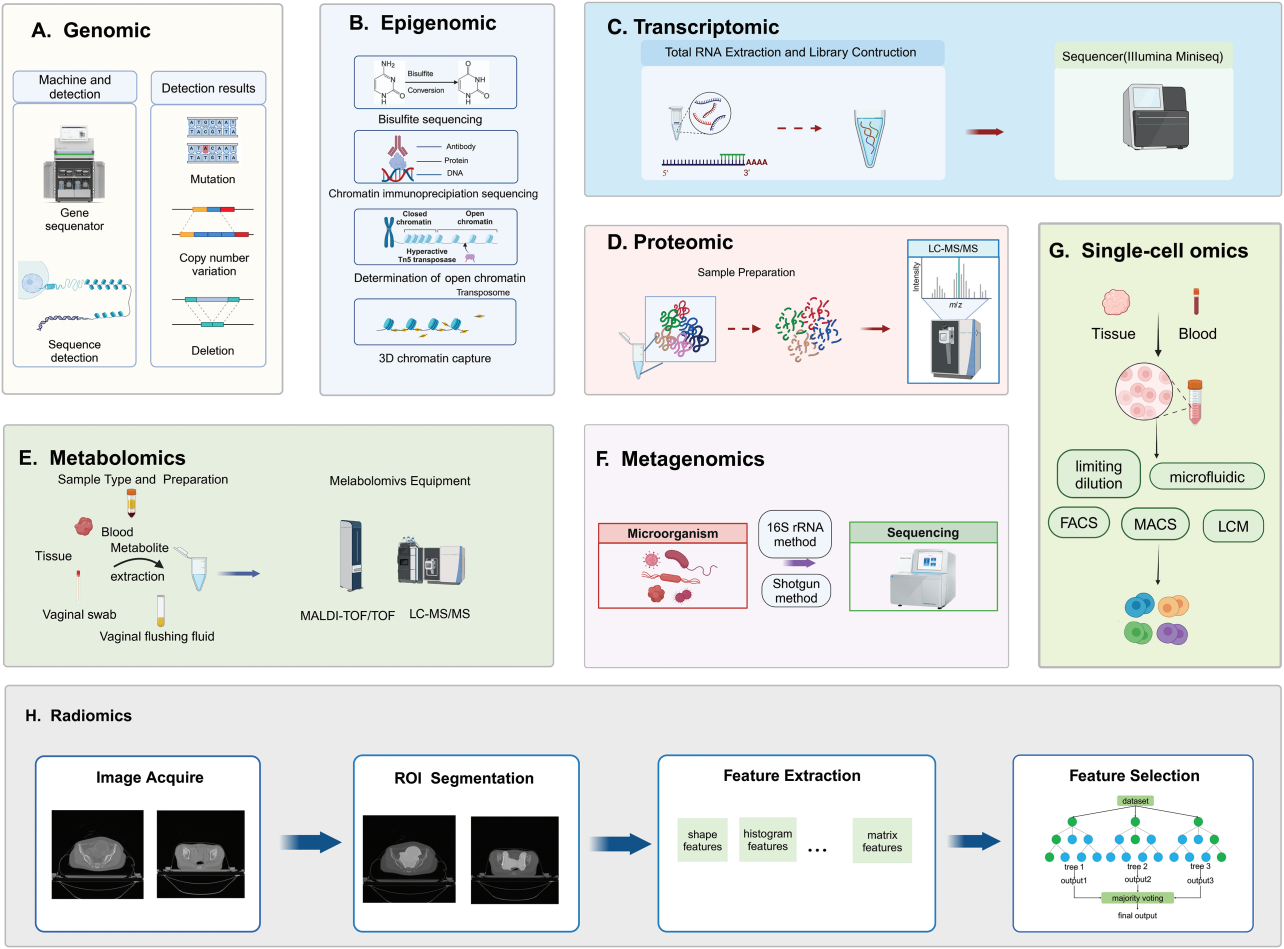
Technical principles of multidimensional omics measurements. A, B, C, F, and G are based on sequencing methods, D and E are based on MS methods, and H is based on imaging methods. (A) Genomics involves DNA extraction, library preparation, sequencing, and detection of genetic variations; (B) Epigenomics sequencing methods include bisulfite sequencing, chromatin immunoprecipitation sequencing, determination of open chromatin, and 3D chromatin capture techniques; (C) Transcriptomics involves total RNA extraction, RNA fragmentation, library preparation, and sequencing; (D) Proteomics involves enzymatic digestion of protein samples, desalting of peptides, and detection using LC-MS/MS; (E) Metabolomics includes sample preparation, instrument detection, and data analysis; (F) Metagenomics, typically utilizes 16S rRNA sequencing and shotgun sequencing methods to analyze microbial communities; (G) Single-cell omics from isolating individual cells from tissue or plasma samples, capturing mRNA, transcribing it into cDNA, amplifying it, and then sequencing it; (H) Radiomics involves acquisition of imaging data, delineation and segmentation of tumor regions, feature extraction, and quantification.

Genomic methods primarily focus on DNA sequencing to identify specific mutations associated with genetic carcinogenesis and analyze chromosomal rearrangements to characterize cancer types or subtypes [13]. Second-generation sequencing, also known as next-generation sequencing (NGS), provides a practical solution to these challenges. NGS systems typically involve five main steps: nucleic acid extraction, library construction, template amplification, sequencing reaction, and data analysis [14]. Single-molecule sequencing (SMS) has advanced by sequencing DNA molecules individually. SMS does not require PCR amplification based on single molecule electrical or chemical signal detection, enabling individual sequencing of each DNA molecule. SMS includes Single Molecule Real Time Sequencing (SMRT) and Nanopore sequencing technology [15]. Whole Genome Sequencing (WGS) and Whole Exome Sequencing (WES) are two primary genomic research technologies. WES can be used to perform NGS of exon-enriched samples and to identify protein-coding mutations. It involves three steps: exome enrichment, high-throughput DNA sequencing, and biological interpretation [16]. WGS sequences the entire genome, providing the most comprehensive analysis of the genome’s spectrum and potential biological consequences. It enables the discovery of new molecular changes in both coding and non-coding regions of the genome [17]. 3D genomics, in contrast to classical genomics, considers the intricate arrangement of chromosomes and the spatial positioning of genes, providing deeper insight into functional, structural regions, and intermolecular interactions within different regions of a three-dimensional framework [18]. Genomics techniques allow for the detection and analysis of pathogenic genes in these tumors, facilitating the identification of tumor subtypes, prediction of prognosis, and genetic risk assessment for patients (Fig. 3A).

In addition to detecting DNA sequences, genomic testing can reveal genetic variations such as gene mutations, copy number variations, chromosomal structural variations, and genotype variations. Genomics-based detection techniques are increasingly employed to guide the application of immunotherapy strategies for patients with gynecological tumors in research and clinical settings. Howitt et al. studied 71 patients with squamous cell carcinoma of the cervix or vulva to explore the link between PD-L1 protein expression and abnormalities in the *CD274* and *PDCD1LG2* genes. They reported a positive correlation between high PD-L1 copy numbers and elevated PD-L1 expression [19]. Similarly, Huang et al. noted that increased *CD274* copy numbers were linked to increased PD-L1 expression in CC [20]. These results on the whole suggest that *CD274* CN changes could be an independent predictive biomarker for the ICPI response. Therefore, it is essential for CC patients receiving PD-L1 immunotherapy to be tested for the coding copy number of key genes. Li et al. classified immune subtypes of CC patients basis of the expression of immune-related genes. Patients with high levels of B cells, CD4^+^ T-cells, and CD8^+^ T-cells exhibited increased somatic mutations and activation of immune pathways, indicating a positive relationship between immune status and mutation burden [21]. This classification system can identify the immune subtypes of cervical squamous cell carcinoma (CSCC) and discover that patients in the immune-enriched subtype may benefit more from ICI, while immune-desert subtype patients could potentially benefit from therapies targeting G protein-coupled receptor (GPCR) and its downstream signaling molecules. The GPCR pathway represents a potential therapeutic target for CSCC. This classification can aid in personalized treatment approaches for patients in clinical settings. One study revealed that CC patients with the *PIK3CA-E545K* mutation exhibited increased PD-L1 transcription and translation compared with those with wild-type *PIK3CA*, leading to reduced CD8^+^ T-cell differentiation in patients with this mutation [22]. Notably, a CC patient with systemic metastasis and the *PIK3CA-E545K* mutation achieved a complete lesion response after receiving only pembrolizumab treatment. The study found that *PIK3CA* mutation alone or in combination with PD-L1 positivity may serve as biomarkers to identify patients with CC who would benefit from immune checkpoint blockade (ICB) therapy. Moreover, in preclinical studies of CC, PI3Kα inhibitors enhanced the anti-tumor efficacy of PD-1 blockade, suggesting their potential as a choice for personalized clinical treatment, warranting further clinical and mechanistic research. Moreover, CD8^+^ T-cell infiltration in tumors is influenced by the *PARP1* gene, with mutations in *PARP1* potentially indicating increased infiltration [23]. In summary, genomic detection methods are increasingly used in CC immunotherapy to identify genetic abnormalities and immune cell statuses, guiding treatment strategies.

### Epigenomics

The concept of “epigenetics”, as introduced by Conrad Waddington, pertains to heritable modifications in cellular characteristics that are not dependent on changes in DNA sequence [24]. Mutations in chromatin-modifying factors and overall alterations in the epigenetic environment not only contribute to the growth of cancer but also offer promising targets for therapeutic strategies. Current epigenetic studies have focused mainly on DNA methylation, histone modifications, nucleosome remodeling, and noncoding RNAs. DNA methylation involves adding a methyl group to the C5 position of cytosine in DNA with the help of DNA methyltransferases to form 5-methylcytosine [25]. These modifications regulate gene expression by recruiting proteins for gene repression or blocking transcription factors from binding to DNA. Histones, essential chromatin components, influence gene expression through chromatin structure modifications and protein interactions [26]. Genetic and epigenetic changes within the TME significantly impact tumor growth, leading to uncontrolled cancer cell proliferation. The close connection between epigenetics and human health emphasizes its potential impact on the discovery of new pharmaceuticals. The most commonly used method is chromatin immunoprecipitation sequencing (ChIP-seq) using specific antibodies against modified histones. After chromatin fragmentation, the protein of interest is immunoprecipitated along with the associated DNA fragments [27]. Cleavage Under Targets and Release Using Nuclease (CUT & RUN) and Cleavage Under Targets and Tagmentation (CUT & Tag) have become the most reliable alternatives to ChIP-seq. In CUT & RUN [28], cells are fixed on concanavalin A-coated magnetic beads to permeabilize the cell membrane, antibodies targeting the desired DNA-binding proteins are added, DNA is fragmented and purified, then DNA is extracted for library construction and sequencing. Further development led to the CUT & Tag technology [29], which allows direct attachment of sequencing adapters to the cleaved DNA, eliminating the need for library preparation. Alterations in DNA methylation within tumor suppressor gene promoters, characterized by widespread hypomethylation and specific hypermethylation, are significant risk factors for CC development [30]. Consistent promoter hypermethylation silences tumor suppressor genes (TSGs) in human cancers, indicating that DNA methylation is a potential target for early cancer detection and patient prognosis assessment (Fig. 3B).

Considerable focus has been placed on the PAX1 gene, highlighting its methylation levels as essential biological markers for the development of CC and the effectiveness of treatments. These markers are relevant for diagnosing cervical intraepithelial neoplasia [31], detecting invasive CC [32], and predicting responses to concurrent radiotherapy and chemotherapy in CC patients [33]. CSCC, the predominant histological subtype comprising approximately 90% of all CC cases, is characterized by the oncogenic transformation of epithelial tissue cells and squamous cells. In a groundbreaking study, Yu et al. utilized a noninvasive histopathological approach to investigate the methylation status of patients with CSCC, providing significant insights for forecasting clinical outcomes and anticipating treatment responses [34]. This study analyzing gene methylation data identified 14 key genes related to clinical outcomes in individuals with CC. These genes were used to construct a robust model for predicting treatment responses in individuals with CC. Specifically, the risk model utilizing methylation patterns was constructed, revealing correlations with immune system biomarkers such as PD1 and CTLA4. Additionally, the integration of whole-slide imaging (WSI) for the analysis of tissue sections led to the creation of a prognostic model for patient survival utilizing imaging data. The construction of this model is based on the pathological features driven by methylation of genes. Patients with this characteristic in CC can be distinguished in terms of clinical outcomes, tumor infiltration status, and effectiveness of immunotherapy. This could enhance patient management and promote personalized treatment strategies.

### Transcriptomics

Transcriptome analysis examines gene expression profiles by focusing on all RNA molecules in cells, cell types, or organisms. The sequencing and analysis of RNA molecules can reveal gene expression levels, splice variations, and transcriptional regulation, aiding in the detection of gene expression changes and interpretation of the TME in cancer research [35]. Transcriptome analysis, including alternative splicing assessment, identification of fusion transcripts, noncoding RNA exploration, and discovery of novel transcripts, is a valuable tool for understanding cancer mechanisms, identifying therapeutic targets, conducting prognostic assessments, and identifying biomarkers [36]. High-throughput methodologies such as microarray and RNA sequencing are commonly used in transcriptome analysis. Microarrays are cost-effective but can only assess genes with known sequences. Traditional RNA-seq, also known as Bulk RNA-seq, is used to measure gene expression patterns, isoform expression, alternative splicing, and single nucleotide polymorphisms [37]. Bulk RNA-seq enables more sensitive and specific detection of transcripts, allowing for more quantitative analysis of differentially expressed genes and detection of genes expressed at lower levels. Bulk RNA-seq is applied at the tissue and cell population levels, suitable for comprehensive transcriptomic sequencing. However, Bulk RNA-seq measures the average expression levels of genes in tissue and does not explore detailed structural and functional differences in cell responses [38]. RNA-seq can capture various types of RNA, including messenger RNA, microRNA, long non-coding RNA, and circular RNA [39]. RNA-seq can reveal the precise locations of transcription boundaries at single-base resolution, uncover sequence variations within transcriptional regions, and also provide all essential information about cellular activities [40]. As the importance of the TME becomes more widely acknowledged, the utilization of transcriptomics in gynecological tumors is increasing (Fig. 3C).

The sensitivity of tumor cells to radiotherapy may be modulated by the TME, which in turn can be altered by the biological effects of radiation [41]. A study by Feng et al. investigated the resistance of cervical carcinoma to radiation via gene expression microarrays [42]. The results showed a strong link between Biological Process enrichment and radioresistance in cervical neoplasms, suggesting that a synergistic interaction between radiotherapy and the immune system affects their effectiveness. The genes ZAP70, CD163, CD247, and CD8B have been identified as signature genes for predicting CC sensitivity to radiotherapy and are associated with the TME, and are potential new treatment targets for CC. Li et al. [43] conducted a study on the correlation between ACSS2, a conserved nucleosidase that converts acetate to acetyl-CoA for energy production, and clinical prognosis as well as tumor immune infiltration in CSCC. By analyzing paraffin-embedded tissues from 240 CC patients using tissue microarrays and referencing the Tumor Immune Estimation Resource (TIMER) database, they found that ACSS2 expression in CSCC is associated with tumor infiltration of B cells, CD4^+^ and CD8^+^ T-cells, and cancer-associated fibroblasts. High ACSS2 expression in CSCC was linked to a shorter OS, suggesting that ACSS2 could serve as a potential prognostic biomarker for CSCC. Furthermore, the development of ACSS2 inhibitors might interfere with immune cells as a therapeutic strategy. Li et al. [44] used TIMER and UALCAN databases to study the mRNA expression and methylation data of Nuclear Factor Erythroid 2-related Factor 2 (NFE2L2) in CSCC using The Cancer Genome Atlas (TCGA). They utilized TIMER and the Tumor-Immune System Interactions Database (TISIDB) to analyze the correlation between NFE2L2 and CD163, focusing on co-expressed genes in tumor-infiltrating immune cells. Data analysis revealed a negative correlation between NFE2L2 and macrophage CD163 expression levels. This study identified NFE2L2 as a potential prognostic biomarker associated with CSCC and related immune infiltration. Wang et al. [45]. conducted a prognostic analysis using transcriptome and clinical follow-up data retrieved from TCGA database for CC. They employed Least Absolute Shrinkage and Selection Operator with Cox Regression Analysis (LASSO-COX) analysis to establish a lncRNA risk model associated with prognosis and copper apoptosis. Tumor Immune Dysfunction and Exclusion (TIDE) was used to assess immunotherapy response, showing lower TIDE scores in low-risk patients with increased checkpoint expression, indicating a stronger immunotherapy response. The risk model includes six lncRNAs related to copper apoptosis, demonstrating efficacy in OS, immune cell infiltration, and immune checkpoint and drug sensitivity, potentially offering new insights for personalized treatment of CC.

### Proteomics

Proteomics is a discipline that comprehensively studies proteins within biological systems [46]. The field aims to understand the structural, functional, and interactive properties of proteins, along with their expression profiles, posttranslational modifications (PTMs), and turnover rates. Proteomic research employs various techniques, with a shotgun being a common method. Shotgun proteomics identifies complete proteins by directly detecting peptide segments obtained from protein enzymatic hydrolysis. The most commonly used method in shotgun proteomics is to digest proteins in a mixture, then separate the peptide segments by liquid chromatography, and then use tandem mass spectrometry to identify the peptide segments, matching them with theoretical peptides in the database for protein identification [47]. Additionally, alternative proteomic methods, such as top-down proteomics for intact protein analysis and middle-down proteomics for larger peptide fragment analysis, provide further insights into protein composition and behavior. MS is a powerful analytical technique widely used in proteomics for high-throughput identification of many proteins, even with limited clinical samples [48]. Matrix-assisted laser desorption/ionization (MALDI) and electrospray ionization (ESI) are widely employed soft ionization methods in MS-based proteomics. MALDI combined with Time of flight (TOF) mass spectrometry is utilized for MALDI-MS imaging, enhancing spatial resolution and rapid data acquisition [49]. Typical detection procedures involve protein digestion, analysis of peptide fragments using MALDI-TOF MS, and comparison of mass fingerprints or amino acid sequences with databases for protein identification [50]. ESI allows for database searching of tandem mass spectra to identify proteins based on their amino acid compositions. For example, in our research group, Han and colleagues thoroughly reviewed nucleic acid and virus detection, laying the groundwork for exploring potential MALDI-TOF MS-based analytical strategies for clinical analysis and research [51]. Additionally, Han et al. devised an enrichment strategy characterized by high selectivity and specificity for validating phosphorylated peptides using MALDI-TOF MS [52]. Moreover, owing to its high level of automation and integration, after separating clinical samples with LC-ESI-MS, real-time ionization enables the identification and assessment of proteins and their posttranslational modifications, especially in the context of CC (Fig. 3D).

Proteomic analyses have identified disease biomarkers and treatment targets, enhancing our understanding of cancer progression and therapy. HPV is the most important single pathogen in CC, mainly contributing through viral oncogenic proteins E6, E7, and E5. Hao et al. conducted a proteomic analysis of cervical exfoliated cells and paired serum samples from patients with HPV-induced CC and precancerous lesions. Candidate biomarkers were evaluated using ELISA and parallel reaction monitoring MS. They found that after CRISPR/Cas9 gene editing depleted E6/E7 in KoE6/E7 SiHa cells, levels of ANT3 and FBLN1 were downregulated, suggesting their expression in CC may be influenced by HPV infection. FBLN1 and ANT3 could serve as universal serum protein markers for CC and HPV infection, potentially enabling the development of an FBLN1 Enzyme-Linked Immunosorbent Assay (ELISA) kit for early detection of CC [53]. The TME undergoes complex changes during different stages of CC progression. Liu et al. conducted a proteomic analysis using label-free quantitative MS on tissue samples from stage I and II CC patients [54]. This study revealed that proteins related to cell growth and intercellular matrix development are more abundant in the TME of stage II CC patients than in the TME of stage I CC patients. Multiple collagen proteins, such as COL12A1, COL5A1, COL4A1, and COL4A2, were notably upregulated in stage II CC patients, indicating their importance in the detection of cancer progression. This study offers valuable insights for early cancer detection and the development of therapies to address the immunosuppressive tumor environment.

### Metabolomics

Metabolomics primarily focuses on downstream effects on gene, RNA, and protein expression. It can detect subtle changes in organisms not captured by other omics methods [55]. Metabolites are sensitive indicators of cellular processes, as minor changes in protein expression or structure can lead to significant alterations in metabolite levels. Additionally, metabolite changes can signal variations in enzyme and protein functions [56]. Intermediate metabolites such as lipids, amino acids, and nucleotides are crucial for cellular processes such as inflammation, proliferation, and migration and are closely linked with immune evasion [57]. Metabolomics measurements employ a wider range of instruments compared with proteomics and genomics because of the numerous unique chemicals present among metabolites, spanning multiple chemical classes. Nuclear magnetic resonance (NMR) spectroscopy and MS techniques are crucial for metabolomics analysis. High-resolution magic angle spinning (HR-MAS) NMR spectroscopy enables quantitative analysis of metabolites in a safe, non-destructive manner with minimal sample preparation. Commonly used gas chromatography-mass spectrometry (GC-MS) or liquid chromatography-mass spectrometry (LC-MS) is more suitable for detecting easily ionizable compounds, while ion suppression commonly found in complex, heterogeneous mixtures further weakens ionization effects. The complexity of metabolites, with tens of thousands of distinct chemicals, presents challenges for comprehensive detection through a single method. To address this challenge, researchers have integrated diverse analytical instruments for CC research based on specific experimental goals. Wang suggested a method to annotate and quantify metabolites in biological samples by aligning ions using a spectral-stitching DI-nESI-HRMS approach in the DIA mode [58]. Metabolomics can systematically identify and quantify all metabolites in biological samples, providing essential information about the cancer status that other omics technologies cannot offer [13]. Metabolomics aids in exploring disease progression and manifestations by investigating environmental factors in gynecologic cancer (Fig. 3E).

Compared with serum or tissue samples, vaginal washings, and swabs are more commonly used to assess metabolic profiles in clinical settings because of their noninvasive nature. The main factor linked to cervical cancer development is persistent infection with HPV. A study conducted in 2019 analyzed metabolic characteristics using LC-MS in three patient groups: HPV-negative/positive status, low-grade cervical intraepithelial neoplasia, and high-grade cervical intraepithelial neoplasia or CC [59]. The identification of three-hydroxybutyrate, eicosenoate, and oleate in distinguishing cancer patients from other groups with high accuracy, particularly in patients with high-grade cervical intraepithelial neoplasia dominated by non-Lactobacillus species, results in metabolic disruptions of amino acids and nucleotides. The complex virus-host-microbiota interactions in the cervical-vaginal microenvironment generate unique metabolic fingerprints, which could be utilized for future developments in diagnosis, prevention, or treatment, thereby positively impacting women’s health outcomes. The cervical-vaginal microbiota plays a significant role in the development of CC associated with HPV infection. Zhang et al. [60] conducted metabolomics analysis on cervical-vaginal secretions and serum samples from HPV-infected patients, identifying HPV-specific biomarkers. Their study identified 9,10-DiHOME, α-linolenic acid, ethylparaben, and glycocholic acid as potential biomarkers for HPV infection. This research helps uncover the relationship between cervical microbiota and serum metabolite changes in HPV infection, facilitating early screening and treatment initiation for CC caused by HPV, thereby preventing further tumor progression. Cervicovaginal metabolomics analysis holds promise as a diagnostic, preventative, and therapeutic tool with significant implications for human health.

### Metagenomics

Metagenomics is a research method for the study of the genomes of microbial populations in the environment through high-throughput sequencing [55]. It is used to analyze the diversity, composition, and gene content of microbial communities [61]. In the past, metagenomics relied on traditional culture methods for biodiversity analysis. The typical workflow includes DNA extraction, library preparation, and sequencing on a platform to minimize sequence biases and artifacts. The initial analysis focuses on quality control of sequence reads, removing adapter sequences, low-quality base calls, and contaminant sequences not originating from the source environment [55]. Dysbiosis of the gut microbiota can activate Toll-like receptors, inducing DNA damage and metabolic/hormonal dysregulation, affecting gastrointestinal tract health and influencing the prevention, treatment, and prognosis of gynecological tumors [62]. Studying the connection between the metagenome and the immune microenvironment of gynecological tumors is crucial. The microbiota in the female reproductive tract plays a significant role in reproductive health and gynecological cancers [63]. Most microorganisms in the female reproductive tract (FRT) are present exist in the vagina and are affected by age, hormones, and hygiene habits. In healthy women of childbearing age, the vaginal microbiota is usually less diverse and is mainly dominated by one or more Lactobacillius species. These lactobacilli help maintain an acidic vaginal environment and act as a protective barrier against harmful microorganisms [64]. Nevertheless, alterations in the microbial composition associated with certain gynecological cancers may significantly deviate from this norm and may serve as predictive indicators for the onset and progression of cancer. With its high-throughput sequencing, metagenomics can be used to analyze microbial genomes to improve the understanding of disease development, aiding in monitoring and immunotherapy selection for CC patients (Fig. 3F).

This research, which is consistent with the literature, indicates that changes in Lactobacillus diversity and abundance within the microbiota may predispose individuals to high-risk cervical dysplasia (HRCD). This finding underscores the significance of the metagenome in shaping the immune microenvironment in CC, which is crucial for managing gynecological malignancies. In this study, they used 16S rRNA amplicon sequencing to analyze the vaginal microbiota in women with HRCD and healthy controls. They found a marked increase in microbial diversity in the HRCD group compared with that in the control group, which was consistent with previous research. Additionally, Firmicutes was identified as the predominant phylum in both groups, with a lower abundance in the HRCD group than in the healthy control group. They successfully constructed a diagnostic model for HRCD, biomarkers from the vaginal microbiota have the potential to serve as non-invasive diagnostic tools for HRCD, enabling more precise treatment strategies for CC patients [65]. Alterations in the microflora may potentiate inflammatory reactions by activating microbial-associated molecular patterns (MUAPs) and their corresponding pattern recognition receptors (PRRs), resulting in increased expression of proinflammatory cytokines such as interleukin-17 (IL-17) and tumor necrosis factor-α (TNF-α) [66]. Li et al. utilized 16S rRNA sequencing to explore the associations among the vaginal microbiome, immune factors, and CC development [67]. Compared with healthy controls, CC patients had reduced levels of Lactobacillus and increased levels of Prevotella and Gardnerella. Furthermore, increased levels of inflammatory immune factors such as interferon-γ (IFN-γ)-induced protein 10 (IP-10) and VEGF-A were detected. These results indicate the vaginal microbiota and specific immune factors may represent a potential non-invasive and straightforward method for predicting CC. Furthermore, VEGF impedes dendritic cell maturation and promotes tumor immune evasion, fostering a favorable microenvironment for CC development. Several studies have assessed the impact of the gut microbiome on the prognosis of CC patients. Sims et al. noted significant differences in microbial enrichment between short- and long-term survivors. Moreover, patients with higher microbiome diversity showed increased CD4^+^ lymphocyte tumor infiltration and activation of CD4^+^ T-cell subsets expressing ki67^+^ and CD69^+^ during radiotherapy [68]. Consequently, the gut metagenome could be a prognostic indicator of survival for CC patients undergoing radiotherapy and chemotherapy. Modulating the gut microbiota before radiotherapy or chemotherapy may offer another approach to enhance treatment efficacy and improve outcomes for CC patients. Mitra et al. discovered that balancing the gut microbiota may enhance immune adaptability and reduce gastrointestinal toxicity after pelvic radiation therapy in CC patients [69]. Łaniewski et al. detected multiple immune checkpoint proteins in the TME and found that four immunosuppressive checkpoint molecules (PD-1, HVEM, LAG-3, and TIM-3) and two immunostimulatory checkpoint molecules (CD27 and CD40) were markedly increased in the CC group. Additionally, the CD40, CD27, and TIM-3 immune checkpoints exhibit unique differences in CC. PD-L1 and LAG-3 levels are negatively correlated with Lactobacillus abundance, while TLR2 is positively correlated. The interconnection of multiple immune checkpoint proteins is linked to cancer features, inflammation, and the microbiota [70]. Additionally, the CD40, CD27, and TIM-3 immune checkpoints exhibit unique differences in CC. PD-L1 and LAG-3 levels are negatively correlated with Lactobacillus abundance, while TLR2 is positively correlated. The interconnection of multiple immune checkpoint proteins links cancer features, inflammation, and the microbiota. Currently, our research group is conducting metagenomic analysis to evaluate the effectiveness of immunotherapy for recurrent and metastatic CC. By studying the intestinal and vaginal microbiomes at different intervals and using a human intestinal microbial ecosystem simulator, we aimed to offer valuable insights into immunotherapeutic strategies for treating CC.

### Single-cell omics

Tumors exhibit significant heterogeneity between cancer cells and TME, featuring diverse cell populations with varied responses to treatment. Traditional cancer omics methods struggle to fully capture this cellular-level heterogeneity and variability [71]. Single-cell technologies offer a promising approach for profiling the landscape of a single-cell atlas at a high resolution, revealing biological insights inaccessible through bulk omics analyses. Currently, single-cell omics, especially single-cell transcriptomics, are widely used in gynecological tumor research and have yielded significant findings. Its crucial role is to dissect the complex TME and guide the application of immunotherapy for gynecological tumors. Advancements in single-cell isolation methods, automation, cost-effectiveness, and increased throughput have greatly enhanced the sensitivity, accuracy, and efficiency of single-cell transcriptomics. In gynecological tumor studies using single-cell transcriptomics, peripheral blood, and surgically removed tissue are commonly used samples. Single-cell omics involves amplifying minute amounts of whole-genome DNA, mRNA, proteins, and metabolites from isolated individual cells, thereby revealing genetic information about cellular heterogeneity. The initial step in single-cell omics is isolating individual cells from tissues. Common methods for single-cell isolation include mouth pipetting, serial dilution, robotic micromanipulation, flow-assisted cell sorting (FACS), and microfluidic platforms [72]. Consequently, selecting an appropriate method for preparing single-cell suspensions from tissues is crucial and should be based on the specific circumstances of the study [73]. Advances in single-cell transcriptomics and related omics technologies have greatly enhanced research on the immune microenvironment of gynecological tumors (Fig. 3G).

Immunotherapy shows promise for treating CC by activating immune function. However, its effectiveness is limited by tumor heterogeneity, the complexity of the TME, and a limited understanding of lymphatic metastasis mechanisms in CC. Single-cell omics research has the potential to significantly impact the field. Through their investigation of tumor and normal adjacent nontumor (NAT) tissue from CC patients using single-cell transcriptomic analysis, Li et al. revealed the extensive heterogeneity of human CC cells and identified cancer-associated fibroblasts (CAFs) that could play a role in the progression of CC [74]. Additionally, they identified four distinct subtypes of CC—hypoxic, proliferative, differentiation, and immunoreactive—the stratification of CC tumors not only enhances our understanding of their pathogenesis but also accelerates the development of personalized treatment strategies for CC patients, offering the potential for more precise prognosis and treatment [74]. Moreover, the TIME is intricately linked to therapeutic outcomes and prognostic indicators for individuals diagnosed with CC. Cao et al. conducted a study utilizing single-cell RNA and T cell receptor (TCR) sequencing techniques, resulting in comprehensive mapping of the immune landscape within CC patients [75]. The findings revealed a significant enrichment of T and NK cells in the TME, which transitioned from a cytotoxic phenotype to a depletion phenotype. Notably, cytotoxic large clone T-cells were identified as pivotal effector cells in the antitumor response [75]. The presence of lymph node metastasis (LNM) in CC patients is a significant prognostic factor and plays a crucial role in guiding treatment decisions. In a study by Li et al., single-cell transcriptomics analysis was conducted on primary tumors, metastatic lymph nodes (LNs), and normal LN tissues from four CC patients [76]. This study validated MRC1 as a marker for macrophages in metastatic LNs, suggesting its use as a biomarker for future tumor therapy. These findings also emphasized the role of CAFs in immune response modulation within metastatic LNs. Single-cell transcriptomic research in CC offers significant insights into tumor heterogeneity, the complexities of the TME, and lymphatic metastasis pathways. These findings could advance personalized diagnostic and therapeutic strategies in clinical practice.

### Radiomics

Radiomics techniques provide a noninvasive way to capture intratumoral heterogeneity using imaging modalities such as magnetic resonance imaging (MRI), computed tomography (CT), and positron emission tomography (PET). In 2012, Lambin et al. introduced radiomics to extract multiple image features from medical images for better information retrieval [77]. Radiomics involves five main steps: acquisition and screening of medical imaging data, manual or automatical segmentation of regions of interest (ROIs), and extraction of ROI features using mathematical algorithms. After eliminating extraneous variables using machine learning algorithms, feature dimensionality is reduced. Next, a model is developed and implemented to address specific clinical issues. Before radiomics, imaging characteristics were traditionally assessed and described qualitatively by radiologists or nuclear medicine specialists. Popular machine learning algorithms such as support vector machines (SVMs), random forests (RFs), and logistic regression (LR) were commonly used [78]. Nonetheless, these subjective assessments are prone to significant variability within and between observers. This technology has shown the ability to reveal tumor details that are not easily visible through visual inspection. Currently, radiomics holds promise in CC research, mainly in terms of patient classification and prognosis evaluation using event-time analysis [79]. These efforts aim to increase diagnostic accuracy and guide clinical decision-making, but further prospective studies are needed to validate the efficacy and utility of radiomics in clinical practice (Fig. 3H).

The management approach for CC patients is tailored according to the tumor stage and lymph node status. Research has shown a correlation between splenic 18F-FDG uptake and systemic inflammatory markers. De Jaeghere et al. assessed the spleen-to-liver standard uptake value ratio (SLR) in 18F-FDG PET/CT scans of CC patients, integrating these findings with survival outcomes, treatment responses, tumor immune infiltration, and baseline characteristics [80]. The authors utilized univariate and multivariate Cox regression models to assess the prognostic value of splenic 18F-FDG uptake in predicting outcomes and radiotherapy in patients with locally advanced CC and its association with immune status. Some studies have suggested that distinct subregions exhibit varied responses to treatment or contribute to disease progression [81]. In contrast to existing PET/CT radiomic studies that typically analyze tumors as a whole, Mu et al. developed a model that incorporates radiomic features from distinct subregions with unique metabolic profiles [82]. Radiomic features were extracted from the images, and the LASSO Cox regression method was used to identify the most significant predictive features with nonzero coefficients. By generating radiomic features using a linear combination weighted by the associated coefficients, researchers can predict the PFS and OS of patients with locally advanced CSCC who are receiving concurrent chemoradiotherapy, radiomic features can serve as predictive and prognostic biomarkers for radiotherapy and chemotherapy in patients with locally advanced CC. Currently, there is a temporary dearth of scholarly articles in the field of CC that employ radiomics to investigate the TIME. However, there is a wealth of research on concurrent chemoradiotherapy, with numerous studies demonstrating a strong correlation between radiotherapy and the TIME. Radiotherapy-induced immunogenic cell death (ICD) triggers the upregulation of extracellular calcium-binding proteins, leading to the release of damage-associated molecular patterns (DAMPs), such as high mobility group box (HMGB)1 and adenosine triphosphate (ATP). These molecules can recruit and activate antigen-presenting cells, initiating T-cell responses specific to tumor antigens. Consequently, the potential of exploring the relationship between immune markers and radiotherapy through radiomics is promising. Our research group is presently engaged in a study examining the prognostic implications of immunotherapy for recurrent and metastatic CC utilizing MR imaging. This research effort may provide novel perspectives for assessing the efficacy of immunotherapy for CC.

### Spatial omics

Spatial omics is a rapidly advancing field that maximizes the utilization of spatial structures to further reveal the positions of biomolecules within cells and tissues, shedding light on the spatial relationships of cells and tissues in disease contexts like CC. Currently, Spatial transcriptomics (ST) and Spatial proteomics (SP) are extensively studied in this area. ST enables high-throughput spatial localization analysis and transcriptomic analysis in biological systems for various applications. ST technologies mainly encompass three methods: laser capture microdissection (LCM)-based approaches, *in situ* imaging-based approaches, and spatial indexing-based approaches [83]. LCM methods precisely dissect individual cells from tissue sections, providing spatial information about cells. *In situ* imaging-based methods also capture spatial information of cells, with the most common techniques being *in situ* hybridization (ISH) and *in situ* sequencing (ISS). ISH visualizes target molecules using complementary labeled probes in their native environment [84]. ISS involves reverse transcription of mRNA into cDNA, followed by rolling circle amplification and sequencing, where primers amplify cDNA from circular DNA templates [85]. Spatial indexing methods use barcoding for local hybridization of RNA molecules, followed by NGS to quantify gene expression profiles, contrasting with RNA-seq [86]. Compared to RNA-seq, ST aims to reveal the spatial distribution, subcellular localization, and interactions of genes within cells, offering new perspectives on the spatial regulatory mechanisms of gene expression. SP is a technology focused on studying the spatial distribution and localization of proteins within cells and tissues. In SP research, three commonly used complementary methods include: 1. Organelle analysis based on MS includes single-organelle analysis and multi-organelle analysis. Currently, the MS-based methods for multi-organelle analysis achieve high resolution of organelles, proteomic coverage, and accurate classification. 2. Protein-protein interaction studies often use affinity purification-MS, which is antibody-mediated affinity purification based on mass spectrometry. 3. Imaging-based SP provides the opportunity to visualize proteins in their natural cellular environments without the need for cell lysis or organelle isolation prior to proteomic analysis [87]. Unlike traditional proteomics, SP enables the detection of dozens of proteins without compromising spatial information. Spatial omics provide precise spatial coordinates of cellular and molecular spectra at a systemic level, deepening our understanding of the CC environment.

LNM is a crucial factor in CC outcomes and is strongly linked to clinical and pathological features. SP sequencing was used to analyze tissue samples from CC patients with positive and negative pelvic lymph nodes. This study revealed a notable increase in the expression of nuclear speckle-type pox virus and zinc finger protein (SPOP) in the positive group, with higher levels of SPOP associated with a significant decrease in OS and recurrence-free survival (RFS) among patients with CC. Furthermore, SPOP may inhibit the immune microenvironment by promoting PD-1 dissociation from PD-L1, thereby facilitating pLN metastasis in CC, leading to poorer OS and RFS in patients. This finding will expand clinical understanding of CC progression, and SPOP may emerge as a future therapeutic target [88]. Furthermore, Guo et al. [89] used single-cell RNA sequencing and spatial transcriptome techniques to study cell subset transitions from normal cervical tissue to precancerous lesions and identified three distinct clusters: HPV-related normal, High-grade Squamous Intraepithelial Lesion (HSIL), and cancer and identified key nodes that may determine disease progression, further revealing unknown mechanisms of HPV-mediated carcinogenesis. This study may provide new insights into understanding the pathogenesis of CC clinically and also offers new possibilities for precise diagnosis, treatment, and prognosis prediction for patients with precancerous lesions and CC. A study by Ou et al. enhanced the understanding of the immune microenvironment in CSCC. They used scRNA-seq and spatial boosted resolution histological sequencing to analyze cervical samples from 2 noncancerous patients and 14 patients with CSCC. A study revealed that a specific group of myofibroblasts is associated with poorer survival probabilities in CSCC patients, it predicts resistance to immunotherapy, and the spatial distribution and potential functions of myofibroblasts have been validated. These findings suggest that myofibroblasts in the TME may facilitate tumor progression and metastasis by inhibiting lymphocyte infiltration and modulating extracellular matrix remodeling. The study found that combination therapies targeting multiple biological processes would be a better approach for treating CSCC. This research enhances our understanding of the immune microenvironment in CSCC and provides new insights into the treatment of advanced CSCC [90].

## Multi-Omics Approaches

Multi-omics analysis enables a thorough investigation of diverse molecular data across all biological levels, presenting challenges in deriving significant conclusions from the increasing quantity of multi-omics data. Our research group has disseminated numerous articles utilizing multi-omics methodologies to investigate protein-metabolite associations in matched plasma samples [91–93]. These investigations integrate metabolomic and proteomic profiling data, providing a platform to establish connections between circulating proteins and metabolites as collaborative partners in human physiology. Single-cell multi-omics notably improves traditional scRNA-seq and reveals a more thorough and conclusive representation of the TIME by integrating diverse data modalities. In a study that integrated various omics data in the context of CC, machine learning techniques were utilized to investigate the enrichment of tumor-type-specific features. This study proposed a comprehensive approach demonstrating the concurrent relationships among the oncogenome, microbiome modules, and CD8^+^ T-cells or TAM1 cells, emphasizing the potential role of the microbiota in either inhibiting or promoting tumor immune responses [94]. Another notable study examined the influence of microbiomes, vaginal pH, immunoproteomes, and metabolomes in cervicovaginal samples obtained from 72 women in Arizona, with or without cervical neoplasms, utilizing neural networks and random forest supervised learning techniques. Data integration uncovered associations between crucial immune regulators and cancer indicators linked to cervicovaginal health or dysbiosis, as well as the prevalence of bacterial taxa, indicating interrelations among the microbiome, metabolome, and immunoproteome. The authors also revealed MIF as a crucial immune biomarker linked to vaginal microbiota composition and vaginal pH, along with IL-6, IL-10, and MIP-1α, in relation to genital inflammation, emphasizing the complex interplay between the vaginal microbiota and host immune responses [95].

CSCC exhibits a restricted reaction to ICB therapy. Fan et al. conducted a thorough multi-omic investigation involving single-cell RNA sequencing, ST, and SP. The intricate, spatially detailed profiles of intratumoral expression heterogeneity in CSCC were established by integrating genetic and pharmacological interventions. The different phases of squamous differentiation can be distinguished on the basis of the tumor status associated with epithelial-cytokeratin, epithelial-immune (Epi-Imm), and epithelial cell senescence. Furthermore, Recombinant Fatty Acid Binding Protein 5 (FABP5) in Epi-Imm tumors malignant cells engage with T-cells and NK cells in response to interferons. The initial examination of a clinical trial (NCT04516616) focusing on CC suggested that neoadjuvant chemotherapy elicits a modification in the Epi-Imm state, leading to pathological complete remission posttreatment with ICB when neoadjuvant chemotherapy (NACT) is combined with CSCC immunotherapy, the potential induction of an Epi-Imm state offers a novel therapeutic option. These findings provide new insights and treatment strategies for further management of CC patients [96]. Additionally, research has highlighted the significant correlations among the metagenomes, metabolism, and various diseases. HPV has long been recognized as the primary driving factor for CC. With the widespread implementation of CC screening and the adoption of HPV vaccines, it is expected that HPV-related CC rates will decrease. Therefore, the early diagnosis and treatment of HPV-independent CC (HPV-ind CC) are highly important. Wang et al. performed Reverse Transcription-Polymerase Chain Reaction (RT-PCR) and RNA-seq on 1010 CC patients to confirm HPV subtypes, and finally, 25 CC were determined to be HPV-ind. Utilizing WES and RNA-seq was conducted on HPV-ind CC, yielding a characteristic spectral profile. The results revealed that HPV-induced CC is more common in adenocarcinomas, which may also be a reason for the lower survival rate in adenocarcinomas. And indicates the importance of PIK3CA mutations and PI3K pathway activation in tumorigenesis, the PIK3CA mutation is associated with resistance to CC treatment, suggesting the potential significance of PI3Kα inhibitors in HPV-ind CC patients, providing new targets for the treatment of HPV-ind CC [97]. Previous studies have shown that genes exhibiting the connection with the tumor microbiome are predominantly associated with infection-related processes, TP53 transcription regulation, and antigen presentation. Correlation analyses involving CD8^+^ T-cells or TAM-1 cells suggest the potential involvement of Megasphaera in CC and Lottiidibacilli in ovarian carcinoma in tumor immunosuppression. Furthermore, the microbiome genome prognosis model has displayed robust predictive capabilities for short-term prognosis, and the study also found that Tissierella drugs exhibit a certain inhibitory effect on cancer, potentially offering new therapeutic directions for clinical applications, and requiring further research and validation [94]. Owning to the noted connection of tumor angiogenesis and cuproptosis with the TME, a multi-omics analysis was performed to profile the high or low cuproptosis-associated angiogenesis (CuRA) scores using single-cell sequencing dataset and TCGA dataset and construct a CuRA model for prognostic evaluation in CC. The model holds promise for developing innovative prognostic prediction models and treatment methods for CC patients. Additionally, the study found that patients with high CuRA had less CD8^+^ T-cell infiltration, and most of their immune checkpoint genes were reduced, making immunotherapy more difficult in these patients. The study validated an independent prognostic gene, SFT2D1, which is highly expressed in CC and positively correlated with microvessel density. Knockdown of SFT2D1 significantly inhibits the proliferation, migration, and invasion abilities of CC cells. CuRA gene features contribute to developing new strategies for personalized precision treatment of CC patients [98]. A more recent study conducted by the Yuan Group similarly focused on the application of multi-omics strategies for improving the efficacy and prognosis of immunotherapy for CC. In particular, as gamma-delta (γδ) T-cells serve as a key component of the TIME, this study further demonstrated that γδ T lymphocytes could be utilized in the development of immunotherapies for cervical cancer. Specifically, a considerably improved prognosis was observed in CCs with high levels of γδ T-cell infiltration, and they are more adaptable to anti-tumor immunotherapies such as treatments of ICI and Tumor Infiltrating Lymphocyte (TIL). Additionally, noninvasive assessment of γδ T-cell infiltration in CC tissues can be observed by MRI-based radiomics models, laying the foundation for more comprehensive research [99].

## Clinical Application

CC is a complex systemic disease, exhibiting abnormalities at one or multiple levels of omics. Any single omics study is insufficient to elucidate the complex pathogenesis of CC. Multi-omics provides a better understanding of information about a disease, pointing out the direction to discover the causes, consequences, and related interactions of malignant tumors. Multi-omics technologies analyze multiple layers of biology simultaneously, exploring the functions of molecules at different levels in the biosystem together, and revealing the specific mechanism of tumor occurrence and development in a more systematic way. Integrating omics studies overcomes the limitations of single omics analysis, detecting molecular differences that single omics alone cannot perceive. This provides more accurate molecular classification of tumors and more precise predictions of death or recurrence probabilities in cancer patients [100]. Currently, the gold standard for diagnosing CC is still histopathological analysis of biopsy samples or postoperative pathological examinations. However, these methods are invasive, and there is a certain risk of needle tract metastasis with biopsy. Therefore, utilizing multi-omics approaches to conduct in-depth analysis of samples from CC patients has expanded the range of detectable molecular biomarkers facilitating precise clinical diagnosis and early cancer detection while reducing the harm caused to patients by biopsies.

By utilizing multi-omics methods, we can accurately determine the pathological type of the patient, provide a systematic basis for classification, enable personalized treatment by selecting the optimal immunotherapy strategy for each patient, and also enhance the monitoring of internal changes in patients during the process of immunotherapy, optimize treatment outcomes and prognosis. To overcome cancer, integrating multi-omics approaches helps to understand the molecular mechanisms of tumorigenesis, development, and drug resistance and provides a theoretical basis for identifying potential therapeutic targets, immune escape, and resistance mechanisms, thereby enhancing the efficacy of immunotherapy in CC treatment. Tumor malignant evolution involves complex interactions among various elements as well as thorough changes in the TME and the immune system [101]. For the clinic, multi-omics technologies not only provide the fundamental principles of molecular subtyping but also guide the development of individualized intervention which leads to improved prognosis.

## Challenges and Prospects

Precision medicine in clinical settings highlights the importance of using a multi-omics approach for personalized and predictive medicine in complex diseases [102]. This approach can distinguish variability among patients and improve diagnostic accuracy. But there are some challenges that lie ahead.

Large cohort and large-scale studies are needed to obtain reliable and comprehensive results. To improve reliability and minimize external factors, conducting carefully planned experiments with an adequate quantity of high-quality samples is crucial. Analyzing the longitudinal and horizontal dimensions of tumor occurrence and development in CC at various stages and levels can provide valuable insights. Variability in samples from patients at different tumor stages and locations, along with the sampling methodology used, can greatly influence study outcomes [103]. Consequently, comparing conclusions from different studies is complex due to differences in sampling time, location, method, and instrument parameters. Standardized large-scale studies are essential. However, clustering large cohorts with long-term follow-up can be challenging. It poses economic burdens, especially in studies involving single-cell and specialized omics. Conducting extensive research in gynecological oncology omics requires substantial financial resources.

Data processing is a noteworthy step. It is inevitable that missing values will occur in omics data, especially in single-cell omics where a large number of missing values may occur. Statistical and machine learning analyses usually require complete datasets without missing values. Proper handling of any missing data is necessary. Deleting features with any missing values can reduce uncertainty but may also result in significant data loss. The typical approach is to remove features with missing values beyond a specific ratio of the total samples and then impute the remaining missing values. Different imputation algorithms exist, such as zero, K-nearest neighbors, and mean value imputation, but no consensus exists on the best evaluation method for selecting the most suitable algorithm based on specific circumstances. The usage of deep learning in gynecologic oncology is hindered by small sample sizes, especially within individual studies. Researchers frequently combine and analyze data from multiple studies to overcome this limitation, a common strategy in bioinformatics analyses of diseases. However, differences in sampling procedures, processing methods, and detection platforms among studies can introduce technical variations that mask the genuine physiological changes linked to the disease [104]. There is a lack of gold-standard unified post-processing data analysis protocols including normalization, transformation, and scaling to ensure robustness, reproducibility, and comparability across studies [105]. Careful data preprocessing and alignment are essential.

Integrating multi-omics data can offer valuable insights into the mechanisms operating at different biological levels. Strategies for multi-omics integration include early integration and late integration. Early integration involves the concatenation of all omics datasets prior to analysis, and late integration involves independent interrogation of individual datasets followed by comparative analysis [106]. Early integration has the potential to uncover more relationships between variables from the different layers. However, due to the heterogeneity and complexity of omics data, preprocessing before integration is crucial [107]. Integrating multi-omics data in CC research poses challenges such as increased computational burden and potential redundancy. Minimizing redundancy and enhancing computational efficiency are crucial for preserving data integrity. Current multi-omics data integration often focuses solely on the data, overlooking complex biological interactions among biomolecules. Although models based on machine learning and neural networks have been proposed to include these interactions in data analysis, more effort is needed to fully explore these interactions and the interpretability of results in omics analysis [108,109]. Pathological images are crucial for diagnosing and grading CC. Integrating these images with omics data can potentially improve personalized diagnosis and treatment. Pathological images consist of extensive high-resolution image collections, while omics data are characterized by their structured nature. Further research is needed to explore methods for effectively integrating image features and omics data to develop precise diagnostic and treatment models for CC. Additionally, considering factors such as medical history, lifestyle choices, tobacco use, and alcohol exposure in omics data analysis could improve research outcomes.

Despite progress in omics research on gynecological tumors, there is a gap between research advances and their practical use in clinical settings. It is crucial for findings intended for clinical use to be highly reproducible, which is often lacking in studies with small sample sizes. Furthermore, false positives in omics data present a major challenge in interpreting and applying research findings. Further experiments are needed to validate the omics analysis findings. The results must be interpretable, as most omics analysis outcomes are data-driven. Clarifying the biological significance of these analytical results is crucial for improving the understanding of tumor pathogenesis and developing new clinical diagnostic and therapeutic methods. The feasibility of applying these discoveries in clinical practice, considering technical and economic aspects, is essential for successful translation. Analyzing omics data for individual patients is resource-intensive, creating financial and time burdens. Developing user-friendly detection methods from omics analyses can accelerate research translation into clinical practice, improving patient outcomes.

Despite ongoing challenges, omics research has enriched the understanding of gynecological malignancies. As omics technology matures and analysis algorithms develop, we expect multi-omics to enhance our understanding of gynecological tumors. This advancement is crucial for driving progress in precision medicine for these tumors. The evolution of omics technology and algorithms will boost the effectiveness of multi-omics as a tool for expanding our knowledge and improving precision medicine for gynecological tumors.

## Conclusions

A comprehensive approach is crucial for understanding the complexity and multifaceted nature of the immune microenvironment in precision medicine. The increasing importance of precision medicine in clinical practice has highlighted the use of multi-omics strategies in personalized and predictive medicine for complex diseases. In the precision medicine of CC, there is potential to discover more accurate biomarkers for the early prediction of CC, thus reducing the incidence of cervical cancer. Additionally, multi-omics strategies can help determine personalized treatment plans for late-stage CC patients to achieve personalized treatment and reduce the recurrence rate of CC. Omics analysis thoroughly examines biological samples and is key in studying CC. The progress in multi-omics analysis enhances integrative approaches, leading to a better understanding and treatment of immuno-oncology.

## Data Availability

The content, figures, and any information in this manuscript will be made available by the authors, without undue reservation.
